# Comprehensive assessment of primary and secondary low bone mass using dual-energy X-ray absorptiometry and cone beam CT—a cross-sectional study

**DOI:** 10.1093/dmfr/twaf030

**Published:** 2025-04-17

**Authors:** Ioana Ruxandra Poiană, Iulia Florentina Burcea, Silviu-Mirel Pițuru, Alexandru Bucur

**Affiliations:** Faculty of Dentistry, “Carol Davila” University of Medicine and Pharmacy, 050474 Bucharest, Romania; Faculty of Medicine, “Carol Davila” University of Medicine and Pharmacy, 050474 Bucharest, Romania; Department of Endocrinology, National Institute of Endocrinology C. I. Parhon, 011853 Bucharest, Romania; Faculty of Dentistry, “Carol Davila” University of Medicine and Pharmacy, 050474 Bucharest, Romania; Faculty of Dentistry, “Carol Davila” University of Medicine and Pharmacy, 050474 Bucharest, Romania

**Keywords:** osteoporosis, menopause, secondary, cone beam CT

## Abstract

**Objectives:**

The present study examined the potential use of CT panoramic mandibular indices on cone beam CT (CBCT) for the assessment of bone density in patients with primary and secondary causes of low bone mass.

**Study design:**

The study enrolled 104 postmenopausal women and 66 patients with endocrine-related low bone mass (diabetes mellitus, acromegaly, Cushing syndrome), who underwent dual-energy X-ray absorptiometry (DXA) and CBCT scanning. The study assessed the correlation between DXA parameters (lumbar spine, femoral neck, total hip *T*-score, bone mineral density [BMD], and trabecular bone score [TBS]) and CBCT-derived indices (CT mandibular index superior [CTI(S)], CT mandibular index inferior [CTI(I)], and CT mental index [CTMI]).

**Results:**

Significant correlations were found between the CBCT indices and both quantitative (BMD, *T*-score) and qualitative (TBS) measures of bone mass. In postmenopausal women, all 3 CBCT indices showed strong correlations with DXA parameters. In secondary endocrine causes, CTMI and CTI(S) were significantly correlated with TBS scores, and CTMI also showed a significant correlation with lumbar BMD.

**Conclusion:**

The study concludes that CTI(S), CTI(I), and CTMI are valuable for assessing bone density and quality in patients with low bone mass, both in primary and secondary osteoporosis related to diabetes mellitus, acromegaly, and Cushing syndrome.

**Advances in knowledge:**

These findings support the use of CBCT as a useful tool for evaluating bone health in the clinical setting and optimizing dental implant result. It is among the first studies to evaluate bone mass quality and quantity in secondary endocrine causes of low bone mass.

## Introduction

Osteoporosis is a common pathology encountered in medical practice, being around 22 million people with this diagnosis at European level.^1–4^ The clinical implications are fragility fractures, which increase morbidity and mortality.^5^^,^^6^ The risk of osteoporosis and fractures increases with the onset of menopause and advancing age, these 2 factors leading to the onset of primary osteoporosis.^7^

Around 40% of osteoporosis patients also associate secondary causes.^1^ The secondary causes of osteoporosis and bone fragility are numerous, including endocrine, rheumatological, hematological diseases, malignancies, drugs or nutritional deficiencies. Endocrine causes of osteoporosis represent a heterogeneous group of pathologies characterized by bone fragility, in the presence or absence of the diagnosis of osteoporosis based on DXA.^8^ These are represented especially by acromegaly, Cushing syndrome (CS), hypogonadism, primary hyperparathyroidism, and hyperthyroidism.^9–12^ The hormonal excess or deficiency characteristic for each of these pathologies produces specific changes in bone metabolism and is associated with an increased risk of vertebral and/or non-vertebral fractures.^13–16^ In the literature, the prevalence of radiologically detected vertebral fractures (VFs) in these pathologies varies from 10% to 80%, the highest figures being found in patients with CS and those with acromegaly.^10^^,^^14^^,^^17^

Cone beam CT (CBCT) has become an important tool in dentistry, though its role in evaluating low bone mass remains under investigation.^18^^,^^19^ Unlike traditional radiography, CBCT offers 3D imaging with high resolution and a relatively low radiation dose, allowing for detailed examination of bone structure. This role is particularly beneficial in the context of osteoporosis, where accurate assessment of bone density and microarchitecture is essential.^20–22^ By providing a detailed view of trabecular and cortical bone, CBCT can potentially identify early signs of bone loss and structural deterioration.^23–25^ Moreover, CBCT can be valuable in assessing the effects of secondary disorders on bone quality, offering a comprehensive perspective on bone changes in this context.^26^ The patients included performed CBCT for dental pre-implant evaluation.

Among the first assessments of BMD using CBCT measurements that employed mandibular indices and quantified them in postmenopausal women was conducted by Koh and Kim,^21^ using the superior and inferior cortical indices (CTI(S) and CTI(I)). They observed significant differences between normal bone mass and the osteoporotic group (21 postmenopausal osteoporotic women, *P* < .05). Since then, other studies have attempted to confirm their results or to find other indices derived from CBCT to assess bone density in patients at risk of low bone mass.^27^^,^^28^ Most commonly, mandibular measurements on CBCT images are performed in the MF area,^29^ which is not influenced by the masticatory muscles and also has a fixed position.^30^

The purpose of this study was to evaluate the correlations between CBCT mandibular indices and bone density (regarding both quantity and quality of the bone assessed by DXA and trabecular bone score [TBS]), in postmenopausal women and patients with low bone mass due to 3 endocrine secondary causes (CS, acromegaly, and diabetes mellitus, respectively). The following mandibular indices were used, according to the modified Ledgerton’s classification^31^: CT mandibular index superior (CTI(S)), CT mandibular index inferior (CTI(I)), and CT mental index (CTMI).

Identifying patients with low bone mass or affected bone quality and treat them accordingly pre-implantation would be beneficial in optimizing dental implant results.

## Methods

The present study included a group of 66 patients with secondary osteopenia/osteoporosis, with endocrine pathologies with known effect of lowering bone mass (diabetes mellitus, acromegaly, and CS) and a group of 104 postmenopausal women, with primary osteopenia/osteoporosis (type I—postmenopausal or type II—senile osteoporosis) with normal BMD, osteopenia or osteoporosis, with or without specific anti-osteoporotic treatment.

The inclusion criteria were postmenopausal status or known endocrine pathology with bone effects (diabetes/acromegaly/CS), DXA with TBS evaluation, biochemical evaluation, CBCT evaluation. Exclusion criteria were the presence of systemic diseases affecting bone metabolism (neoplasia, osteomalacia, history of rickets, severe renal failure, liver failure, malabsorption disorders); usage of medications interfering with bone density (except for glucocorticoids); or other endocrine diseases affecting bone mass (hyperthyroidism, hyperparathyroidism).

The patients included were candidates for dental implants and they performed CBCT as part of the pre-implantation protocol (frequently used in our country, especially in private practices that offer personalized protocols), CBCT evaluation being commonly used most of the dental clinics. The patients were evaluated in close collaboration with a private provider of dental imaging with expertise in CBCT imaging and respectively, the National Institute of Endocrinology, a public hospital, that attends hundreds of patients with metabolic bone disease yearly.

Written informed consent was obtained from patients before the study. The study was approved by the Ethics Committee of “C. I. Parhon” National Institute of Endocrinology, Bucharest, Romania (no. 4/April 8th, 2021). The enrolment period of the patients was between April 2021 and April 2024, with a maximum distance of 2 months between CBCT and DXA evaluations.

### Image acquisition and analysis

**Figure 1.**
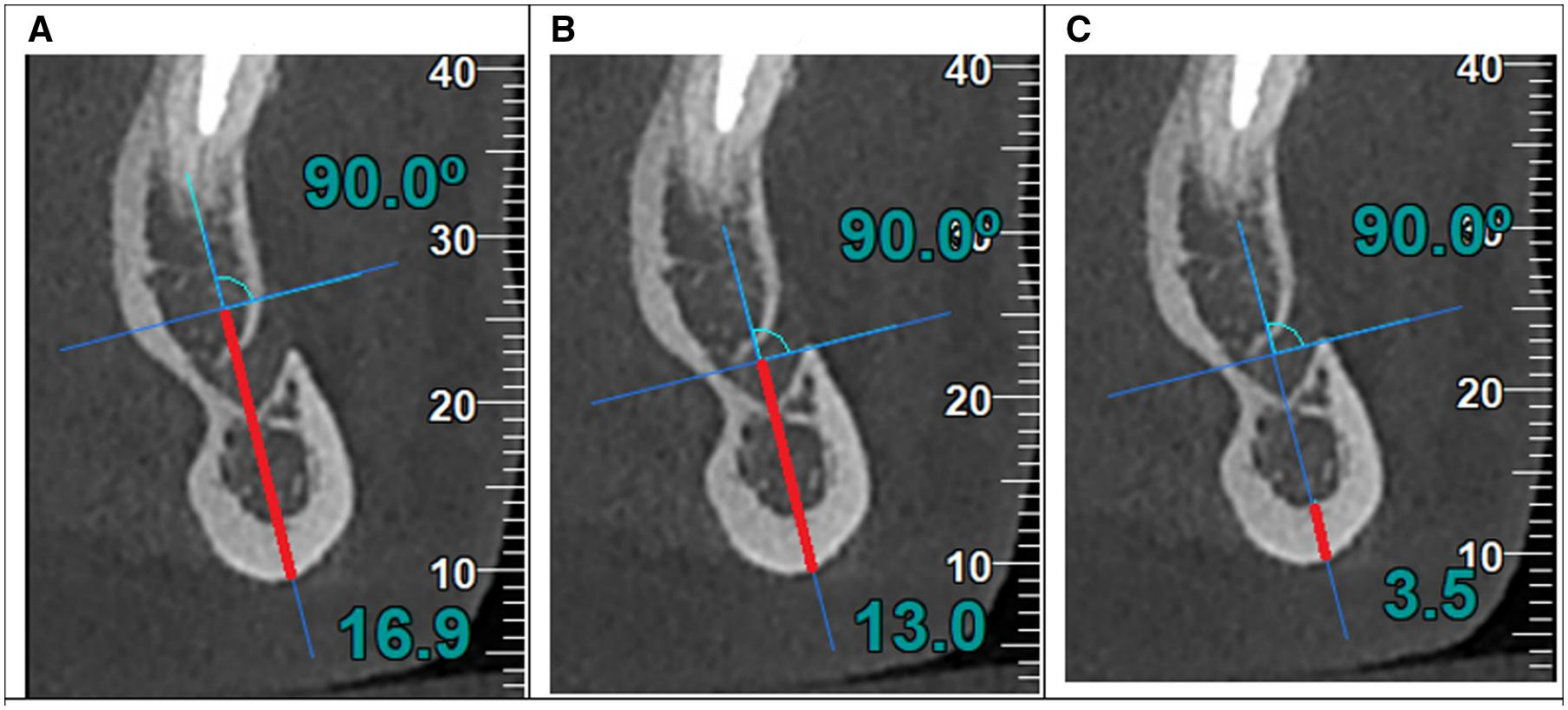
The measurements of S, I, and W indices on coronal CBCT images. (A) S, the distance from the superior margin of the MF to the inferior border of the mandible. (B) I, the distance from the inferior margin of the MF to the inferior border of the mandible. (C) W, the inferior cortical width of the mandible. The indices are the following: CTI(S) = W/S, CTI(I) = W/I, CTMI = W.

The CBCT measurements were performed on coronal images, by an experimented radiologist. The indices are defined, as follows: CTI(S): superior CT mandibular index (the ratio of the inferior cortical width to the distance from the superior margin of the mental foramen to the inferior mandibular border, W/S); CTI(I): inferior CT mandibular index (the ratio of the inferior cortical width to the distance from the inferior margin of the mental foramen to the inferior mandibular border, W/I); CTMI: CT mental index (the inferior mandibular cortical width, W). The W index represents the inferior cortical width of the mandible; the S index represents the distance from the superior margin of the mental foramen to the inferior mandibular border; the I index represents the distance from the inferior margin of the mental foramen to the inferior mandibular border (see Figure 1). The methodology behind these indices are the ones described by Koh et al.^21^

In order to obtain the CBCT images, we used NewTom VGi EVO Cone Beam 3D Imaging (CEFLA s.c.—Via Selice Provinciale 23/a IMOLA, Italy), at 110 kV, 7.5 mA, 3.5 s, pixel size 0.2 mm. For the image reconstruction, we used NewTom NNT (ISDP10003:2020 compliant in accordance with EN ISO/IEC 17065:2012 certificate number 2019003109-2) with Viewer software.

**Figure 2.**
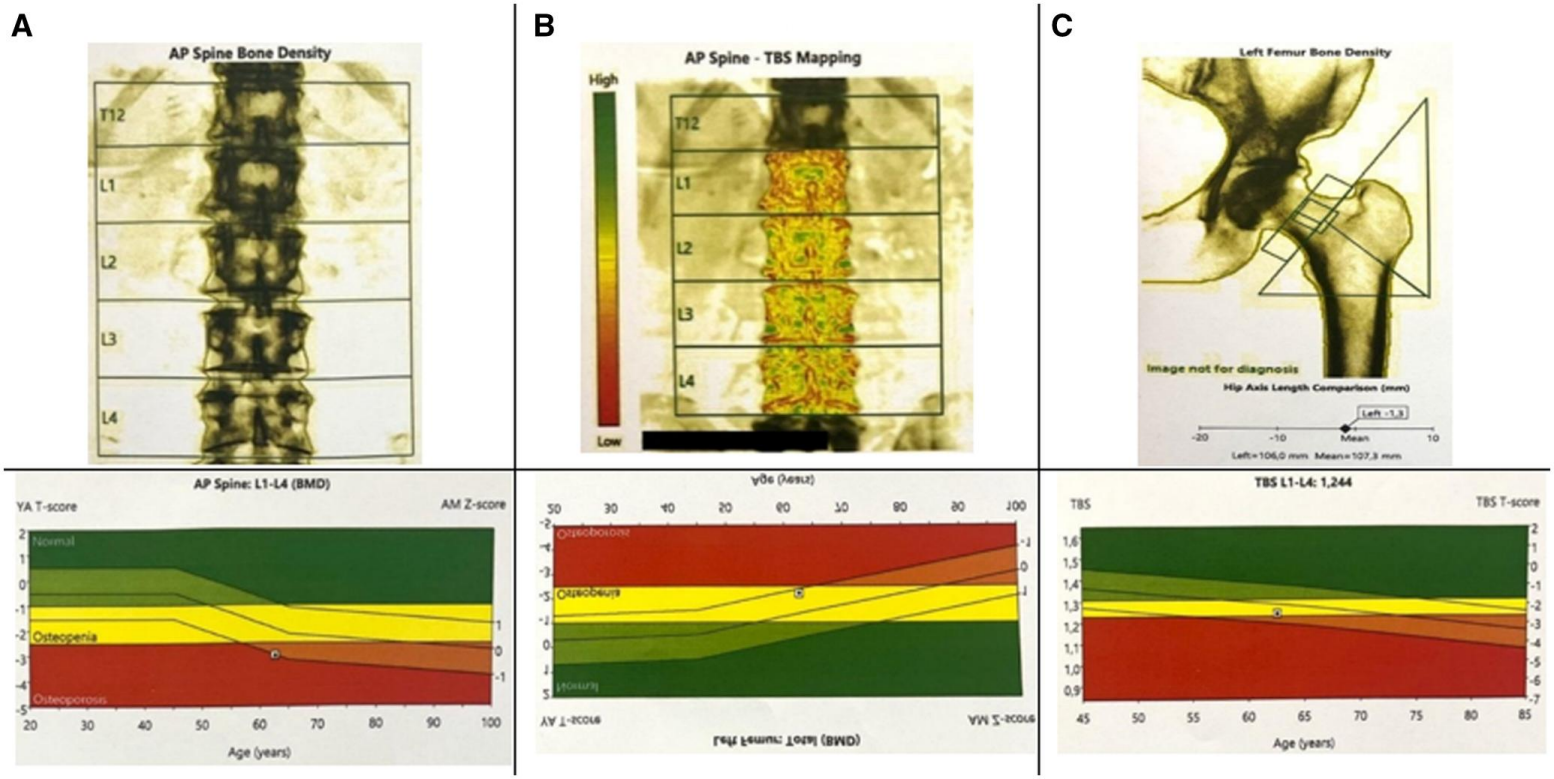
DXA measurements. (A) Spine L1-L4 bone density measurement. (B) Left femur bone density measurement. (C) Spine TBS mapping.

Bone density was measured at the lumbar spine (LS), femoral neck (FN), and total hip by DXA (General Electric Prodigy Lunar, Bedford, UK) using an enCore Software 10 50 086. BMD was expressed in grams per square centimetres (g/cm^2^), and by comparing the BMD with the peak bone mass of a young adult, a *T*-score was obtained, expressed in SD and a *Z*-score for age-matched SD^15^ (see Figure 2). All measurements were done according to the International Society for Clinical Densitometry (ISCD).^32^

TBS values were obtained by analysing the L1-L4 vertebrae DXA images with an iNsight Software version 2.2.0.0 (Medimaps Group SA Headquarters, Switzerland).

All patients were scanned on the same DXA machine, yet by 2 different operators, thus allowing a user bias.

According to the 2020 American Association of Clinical Endocrinologists (AACE) guidelines, the diagnosis of osteoporosis in postmenopausal women is based on the following criteria^33^:

- *T*-score −2.5 SD or below in the lumbar spine, femoral neck, total proximal femur, or 1/3 radius.
- Low-trauma spine or hip fracture (regardless of bone mineral density).
- *T*-score between −1.0 and −2.5 SD and a fragility fracture of proximal humerus, pelvis, or distal forearm.
- *T*-score between −1.0 and −2.5 SD and high FRAX^®^(fracture risk assessment tool) fracture probability based on country-specific thresholds.

### Statistical analysis

We statistically analysed the patients based on the value of the BMD (lumbar spine, femoral neck, total hip scores), *T*-score (lumber spine, femoral neck, total hip scores), respectively, and TBS as continuous values, regardless of the osteoporosis diagnosis at the time of CBCT evaluation.

Alternatively, we used binary logistic analysis to divide the patients based on the osteoporosis diagnosis (according to AACE criteria).^15^ We also employed the regression analysis, parametric tests, the *t*-test, the Pearson’s correlation coefficient, and Spearman’s rho, using IBM SPSS Statistics, version 25 (SPSS Inc, Chicago, IL, United States) for Mac OS.

## Results

The patients’ characteristics from both the groups, postmenopausal women and the 3 secondary endocrine causes that interfere with bone health, divided by a *T*-score diagnostic of osteoporosis based on World’s Health Organization (WHO) criteria (lumbar, femoral and total hip score, less or equal to −2.5 SD)^34^ are listed in Table 1. The study included 104 postmenopausal women, with mean age of 65.15 ± 9.12 years old and a mean age at menopause of 47.22 ± 5.3 years old. The mean lumbar BMD score was 0.944 g/cm^3^ (0.544, 1.437), with a mean *T* lumbar score of −1.95 SD (−5.3, 2.1). For femoral neck, the mean BMD score was 0.944 g/cm^3^ (0.554, 1.221), with a mean *T*-score of −1.95 SD (−3.5, 1.3). The mean score for TBS was 1.284 g/cm^3^ ± 105.83 SD (1.062, 1.558). Regarding secondary causes, the study included 40 patients with diabetes mellitus, 14 patients with CS, and 12 patients with acromegaly.

**Table 1.** Patients’ characteristics.

Parameter	*T*-score ≤ −2.5^a^	*T*-score > −2.5^a^
a. Postmenopausal women		
Number	45	59
Age at menopause (years)	47.18 ± 4.49	47.43 ± 5.73
Femoral neck *T*-score (SD)	−2.06 ± 0.66	−1.15 ± 0.85
Total hip *T*-score (SD)	−1.67 ± 0.78	−0.6 ± 1.04
Lumbar *T*-score (L1-L4) (SD)	−3.12 ± 0.69	−1.3 ± 0.92
Femoral neck BMD (g/cm^2^)	0.727 ± 0.082	0.852 ± 0.11
Total hip BMD (L1-L4) (g/cm^2^)	0.713 ± 0.366	0.941 ± 0.132
Lumbar BMD (g/cm^2^)	0.805 ± 0.088	1.018 ± 0.012
TBS score (g/cm^2^)	1.207 ± 0.181	1.313 ± 0.101
b. Secondary endocrine causes		
Number	26	40
Age at menopause (years)	46.5 ± 54.47	47.4 ± 4.64
Femoral neck *T*-score (SD)	−2.12 ± 0.64	−0.69 ± 1.28
Total hip *T*-score (SD)	−1.56 ± 0.98	−0.18 ± 0.8
Lumbar *T*-score (L1-L4) (SD)	−2.83 ± 0.46	−0.55 ± 1.12
Femoral neck BMD (g/cm^2^)	0.728 ± 0.08	0.913 ± 0.12
Total hip BMD (L1-L4) (g/cm^2^)	0.822 ± 0.14	0.982 ± 0.36
Lumbar BMD (g/cm^2^)	0.815 ± 0.07	1.107 ± 0.17
TBS score (g/cm^2^)	1.288 ± 0.08	1.322 ± 0.142

a Values are expressed as mean ± SD, NOT based on osteoporosis American Association of Clinical Endocrinology (AACE) criteria.

Abbreviations: BMD = bone mass density; TBS = trabecular bone score.

The patients from both included groups showed significantly lower *T-*scores and BMD values in the osteoporosis group compared to the normal/osteopenia group, this being an important reason for comparing these two categories rather than to compare only osteoporosis patients with normal bone mass.^27^^,^^30^^,^^35^ Due to the rich trabecular bone content in the lumbar area, which is more susceptible to bone changes,^36^ the lowest *T*-score observed in the study is for the lumbar spine. This was reported in the context of the postmenopausal women group, where the mean *T* lumbar score was −1.95 SD, but the range extended down to −5.3 SD. The use of all validated DXA sites enhances the depth and understanding of the present results. Assessing bone quality through TBS offers insights into bone quality beyond just bone quantity, making it a valuable tool for evaluating bone health in cases of both primary and secondary osteoporosis.

Lower values of CBCT-derived mandibular indices can be observed in postmenopausal women compared to the secondary endocrine causes group, *P* < .0001. Higher values of CTMI, CTI(S), and slightly higher values of CTI(I) are observed in secondary endocrine causes compared to postmenopausal women. Within secondary endocrine causes, there were also higher values for normal bone quality (TBS ≥ 1.31). Postmenopausal women with normal bone quality also show higher CTMI values (see Table 2). The values of CTI(S) are higher for the patients with intermediate and normal bone quality in both the groups. The mean values for the CMCT indices for both studied groups are shown in Table 2.

**Table 2.** Mean values of the CT parameters on cone beam CT (CBCT) images in postmenopausal women and secondary endocrine causes.

CBCT parameter	Osteoporosis based on AACE criteria^a^	Lumbar *T*-score > −2.5 SD	Lumbar *T*-score ≤ −2.5 SD	Femoral neck *T*-score > −2.5 DS	Femoral neck *T*-score ≤ −2.5 DS	Low bone quality (TBS ≤ 1.23)	Normal bone quality (TBS ≥ 1.31)
a. Postmenopausal women							
CTMI	2.38 ± 0.73	2.74 ± 0.80	2.43 ± 0.83	2.72 ± 0.74	1.84 ± 0.68	2.12 ± 0.61	3.04 ± 0.69
CTI(I)	0.21 ± 0.06	0.24 ± 0.07	0.21 ± 0.07	0.24 ± 0.06	0.17 ± 0.06	0.20 ± 0.06	0.26 ± 0.05
CTI(S)	0.16 ± 0.05	0.18 ± 0.05	0.16 ± 0.05	0.18 ± 0.05	0.13 ± 0.06	0.14 ± 0.04	0.20 ± 0.04
b. Secondary endocrine causes							
CTMI	2.89 ± 0.65	3.03 ± 1.03	2.76 ± 0.54	2.90 ± 0.97	2.77 ± 1.11	2.56 ± 0.88	3.20 ± 1.07
CTI(I)	0.25 ± 0.08	0.27 ± 0.09	0.25 ± 0.07	0.27 ± 0.07	0.24 ± 0.06	0.24 ± 0.07	0.27 ± 0.08
CTI(S)	0.21 ± 0.14	0.24 ± 0.11	0.18 ± 0.047	0.20 ± 0.08	0.17 ± 0.08	0.22 ± 0.15	0.21 ± 0.05

a *T*-score −2.5 or below in the lumbar spine, femoral neck, total proximal femur, or 1/3 radius, low-trauma spine or hip fracture (regardless of bone mineral density), *T*-score between −1.0 and −2.5 and a fragility fracture of proximal humerus, pelvis, or distal forearm, *T*-score between −1.0 and −2.5 and high FRAX (fracture risk assessment tool) fracture probability based on country-specific threshold.

Abbreviations: CBCT = cone-beam CT; CTMI = computer tomography mental index; CTI-(I) = inferior CT mandibular index; CTI(S) = superior CT mandibular index; TBS = trabecular bone score, expressed as g/cm^3^.

In this study, both Pearson and Spearman correlation coefficients were used to analyse the relationship between mandibular CBCT indices and BMD and TBS measurements. The results in Table 3 show significant correlations between CBCT indices (CTMI, CTI(S), CTI(I)) and DXA parameters in both postmenopausal women and patients with secondary endocrine causes of low bone mass. In postmenopausal women, all 3 CBCT indices showed moderate correlations with *T*-scores, BMD, and TBS. In patients with secondary endocrine causes, the CTMI and CTI(S) indices showed significant correlations with TBS scores, and CTMI also correlated significantly with lumbar BMD.

**Table 3.** Correlations between CBCT parameters and bone quantity and quality parameters in patients with secondary causes of osteoporosis versus postmenopausal women.

Parameters for correlations^a^	CTMI	CTI(S)	CTI(I)
a. Pearson’s correlation^a^			
Postmenopausal women			
Lumbar *T*-score^b^	**0.429, *P* < .0001**	**0.387, *P* < .0001**	**0.364, *P* < .0001**
Femoral neck *T*-score^b^	**0.551, *P* < .0001**	**0.465, *P* < .0001**	**0.481, *P* < .0001**
Total hip *T*-score^b^	**0.470, *P* < .0001**	**0.440, *P* < .0001**	**0.451, *P* < .0001**
TBS score^b^	**0.431, *P* < .0001**	**0.421, *P* < .0001**	**0.351, *P* < .001**
Lumbar BMD^c^	**0.359, *P* < .0001**	**0.355, *P* < .0001**	**0.322, *P* < .001**
Femoral neck BMD^c^	**0.522, *P* < .0001**	**0.443, *P* < .0001**	**0.523, *P* < .0001**
Total hip BMD^c^	**0.509, *P* < .0001**	**0.445, *P* < .0001**	**0.481, *P* < .0001**
Secondary endocrine causes			
Lumbar *T*-score^b^	0.205, *P* = .133	0.13, *P* = .33	0.102, *P* = .46
Femoral neck *T*-score^b^	0.116, *P* = .39	0.068, *P* = .62	0.019, *P* = .89
Total hip *T*-score^b^	0.249, *P* = .067	**0.267, *P* = .051**	0.251, *P* = .067
TBS score^b^	**0.411, *P* = .003**	**0.355, *P* = .01**	**0.329, *P* = .021**
Lumbar BMD^c^	**0.298, *P* = .027**	0.20, *P* = .13	0.158, *P* = .25
Femoral neck BMD^c^	0.186, *P* = .17	0.10, *P* = .46	0.054, *P* = .7
Total hip BMD^c^	0.08, *P* = .55	**0.096, *P* = .489**	0.105, *P* = .45
b. Spearman’s rho^a^			
Postmenopausal women			
Lumbar *T*-score^b^	**0.439, *P* < .0001**	**0.386, *P* < .0001**	**0.380, *P* < .0001**
Femoral neck *T*-score^b^	**0.541, *P* < .0001**	**0.439, *P* < .0001**	**0.468, *P* < .0001**
Total hip *T*-score^b^	**0.476, *P* < .0001**	**0.435, *P* < .0001**	**0.449, *P* < .0001**
TBS score^d^	**0.464, *P* < .0001**	**0.460, *P* < .0001**	**0.413, *P* < .0001**
Lumbar BMD^c^	**0.397, *P* < .0001**	**0.371, *P* < .0001**	**0.363, *P* < .0001**
Femoral neck BMD^c^	**0.493, *P* < .0001**	**0.393, *P* < .0001**	**0.416, *P* < .0001**
Total hip BMD^c^	**0.518, *P* < .0001**	**0.450, *P* < .0001**	**0.473, *P* < .0001**
Secondary endocrine causes			
Lumbar *T*-score^b^	0.20, *P* = .125	0.146, *P* = .292	0.121, *P* = .383
Femoral neck *T*-score^b^	0.221, *P* = .106	0.191, *P* = .16	0.133, *P* = .33
Total hip *T*-score^b^	0.247, *P* = .069	0.246, *P* = .073	0.234, *P* = .08
TBS score^d^	**0.446, *P* = .001**	**0.447, *P* = .001**	**0.425, *P* = .002**
Lumbar BMD^c^	0.237, *P* = .082	0.163, *P* = .238	0.140, *P* = .31
Femoral neck BMD^c^	0.237, *P* = .08	0.178, *P* = .19	0.114, *P* = .41
Total hip BMD^c^	0.22, *P* = .1	0.227, *P* = .09	0.233, *P* = .089

a Significant at the .05 level *t*-test (2-tailed) and .01.

b Expressed as SDs.

c Expressed as g/cm^3^.

d Expressed as continuous value.

Abbreviations: BMD = bone mass density; CBCT = cone-beam CT; CTMI = CT mental index; CTI-(I) = inferior CT mandibular index; CTI(S) = superior CT mandibular index; TBS = trabecular bone score, expressed as g/cm^3^.

The logistic regression analysis results in patients with secondary endocrine causes of low bone mass are present in Table 4. Significant associations were found for TBS quality assessment. No association was statistically significant with osteoporosis based on BMD values and AACE criteria for secondary causes. In contrast, for postmenopausal osteoporosis, CTMI, CTI(I), and CTI(S) were effective in predicting bone quality and quantity (*P* < .0001).

**Table 4.** Predictions of osteoporosis and bone quality in secondary causes of low bone mass using logistic regression analysis.

Parameters	Variable	Odds ratio	Model’s sig.
TBS quality assessment^a^	CTMI	1.061, 95% CI (0.997, 1.128)	*P* = .046
Osteoporosis defined as lumbar *T*-score ≤ −2.5 SD^b^		0.957, 95% CI (90.894, 1.023)	*P* = .176
Osteoporosis defined as femoral neck *T*-score ≤ −2.5 SD^b^		0.911, 95% CI (0.790, 1.050)	*P* = .16
Osteoporosis based on AACE criteria^c^		0.97, 95% CI (0.925, 1.018)	*P* = .2
TBS quality assessment^a^	CTI(I)	1.073, 95% CI (0.99, 1.159)	*P* = .006
Osteoporosis defined as lumbar *T*-score ≤ −2.5 SD^b^		0.888, 95% CI (0.756, 1.044)	*P* = .06
Osteoporosis defined as femoral neck *T*-score ≤ −2.5 SD^b^		0.903, 95% CI (0.815, 0.99)	*P* = .54
Osteoporosis based on AACE criteria^c^		0.957, 95% CI (0.906, 1.012)	*P* = .11
TBS quality assessment^a^	CTI(S)	1.115, 95% CI (1.00, 1.243)	*P* = .05
Osteoporosis defined as lumbar *T*-score ≤ −2.5 SD^b^		0.951, 95% CI (0.794, 1.053)	*P* = .2
Osteoporosis defined as femoral neck *T*-score ≤ −2.5 SD^b^		0.823, 95% CI (0.656, 1.032)	*P* = .069
Osteoporosis based on AACE criteria^c^		0.947, 95% CI (0.876, 1.024)	*P* = .169

a Trabecular bone score, expressed as low-intermediate if TBS < 1.31 g/cm^3^ and normal if TBS > 1.31 g/cm^3^.

b Expressed as SDs.

c *T*-score −2.5 or below in the lumbar spine, femoral neck, total proximal femur, or 1/3 radius, low-trauma spine or hip fracture (regardless of bone mineral density), *T*-score between −1.0 and −2.5 and a fragility fracture of proximal humerus, pelvis, or distal forearm, *T*-score between −1.0 and −2.5 and high FRAX (fracture risk assessment tool) fracture probability based on country-specific threshold.

Abbreviations: CTMI = CT mental index; CTI-(I) = inferior CT mandibular index; CTI(S) = superior CT mandibular index; TBS = trabecular bone score, expressed as g/cm^2^.

Table 5 shows the logistic regression analysis for osteoporosis prediction among patients with the three endocrine conditions. The results reflect the variability of the predictive value of CBCT indices across different conditions and bone mass categories. The predictive value of CTMI in acromegaly patients with osteopenia could emphasize the early detection value of this parameter for low bone quality in these patients (Table 5b).

**Table 5.** Predictions between causes of secondary type osteoporosis.

Parameters	Variable	Odds ratio	Model’s sig.
a. Logistic regression for osteoporosis in secondary endocrine causes of osteoporosis			
Diabetes mellitus (*n* = 14) in patients with osteoporosis	CTMI	0.997, 95% CI (0.925, 1.075)	*P* = .94
	CTI(I)	0.987, 95% CI (0.907, 1.073)	*P* = .75
	CTI(S)	1.044, 95% CI (0.984, 1.106)	*P* = .11
Cushing (*n* = 8) in patients with osteoporosis	CTMI	1.084, 95% CI (0.979, 1.201)	*P* = .11
	CTI(I)	1.075, 95% CI (0.96, 1.203)	*P* = .2
	CTI(S)	1.014, 95% CI (0.952, 1.080)	*P* = .68
Acromegaly (*n* = 4) in patients with osteoporosis	CTMI	1.068, 95% CI (0.932, 1.225)	*P* = .33
	CTI(I)	1.000, 95% CI (0.862, 1.161)	*P* = .99
	CTI(S)	0.97, 95% CI (0.815, 1.155)	*P* = .7
b. Logistic regression for normal/osteopenia in secondary causes of osteoporosis			
Diabetes mellitus (*n* = 25) in patients with osteopenia and normal bone mass	CTMI	0.984, 95% CI (0.938, 1.033)	*P* = .51
	CTI(I)	0.976, 95% CI (0.921, 1.033)	*P* = .39
	CTI(S)	0.979, 95% CI (0.9, 1.060)	*P* = .59
Cushing (*n* = 7) in patients with osteopenia and normal bone mass	CTMI	0.989, 95% CI (0.914, 1.071)	*P* = .79
	CTI(I)	0.994, 95% CI (0.906, 1.091)	*P* = .9
	CTI(S)	1.009, 95% CI (0.885, 1.151)	*P* = .89
Acromegaly (*n* = 9) in patients with osteopenia and normal bone mass	CTMI	1.073, 95% CI (1.001, 1.150)	*P* = .045
	CTI(I)	1.046, 95% CI (0.954, 1.148)	*P* = .33
	CTI(S)	1.090, 95% CI (0.953, 1.246)	*P* = .2

Abbreviations: CTMI = CT mental index; CTI(I) = inferior CT mandibular index; CTI(S) = superior CT mandibular index.

## Discussion

Currently, there are no specific recommendations for the diagnosis or treatment of the bone disease from endocrine pathologies, there is only a recommendation for screening bone damage in these patients.^8^^,^^37^ However, not all meet densitometric criteria for osteoporosis, despite having increased bone fragility and a predisposition to develop fragility fractures. This fact is due to the changes given by the hormonal action, especially in patients with acromegaly, who rarely present a densitometric diagnosis of osteoporosis, most often having a BMD similar to that of the general population.^17^ In patients with CS, because of decreased bone quality, fragility fractures may appear even before the significant decrease in bone density.^10^

One of the initial studies to assess BMD using CBCT-derived measurements was conducted by Koh et al,^21^ who focused on mandibular indices in postmenopausal women. They used superior and inferior cortical indices (CTI(S) and CTI(I)) and found significant differences between women with normal bone mass and those with osteoporosis (*P* < .05). This study highlighted the effectiveness of CBCT-derived indices in distinguishing between varying levels of bone mass. The present study assessed the above-mentioned mandibular CBCT indices to validate their reliability in assessing low bone mineral density of secondary causes and compare with the findings from primary osteoporosis.

Following their work, multiple studies tried to confirm those findings or identify additional CBCT-derived indices for evaluating bone mass in patients susceptible of having low bone density.^20^^,^^24^^,^^27^^,^^29^^,^^38^ These studies have validated and expanded upon the initial research, demonstrating the applicability of CBCT measurements in clinical practice for assessing bone health in various conditions, including postmenopausal osteoporosis and endocrine-related low bone mass conditions, such as diabetes mellitus.^24^^,^^39^

Bone densitometry, using dual-energy X-ray absorptiometry (DXA), may underestimate the fracture risk in some chronic diseases (such as glucocorticoid-induced osteoporosis, type 2 diabetes, obesity) and may overestimate the fracture risk in others. One method proposed for bone evaluation is the TBS, which is a technique for determining the alteration of bone quality by indirectly assessing bone microarchitecture at the level of trabecular bone.^40^ A known fact is that diabetic patients have an increased risk of fragility fractures, despite normal BMD values.^41^ Leslie et al^40^ analysed 29 407 women aged over 50 years from the Manitoba cohort and compared them in terms of the presence or absence of a diagnosis of diabetes. No significant differences were observed between the BMD values of the 2 groups of patients, but significantly lower values of TBS in the diabetic ones. During follow-up, patients with diabetes had a significantly higher incidence of major osteoporotic fractures. TBS has been shown to be an independent predictor of fracture risk in both diabetic and non-diabetic patients.^40^ In patients with diabetes mellitus, the present study found that CTI(S) and CTMI showed specific correlations with the TBS, indicating their potential utility in assessing bone quality. The CTMI also demonstrated a significant correlation with lumbar BMD, suggesting its relevance in evaluating bone quantity in diabetic patients.

Regarding acromegaly patients, the effects of excess GH are different on cortical versus trabecular bone.^17^ While cortical bone thickness increases, the effects on trabecular bone are of increased trabecular separation, decreased trabecular thickness, and decreased bone volume/tissue volume (BV/TV) ratio, changes observed in histomorphometric analysis^36^^,^^42^ and also in high-resolution peripheral quantitative computed tomography (HR-pQCT).^9^ Given the specific characteristics of these changes, acromegaly patients have normal BMD values but a higher prevalence and incidence of VFs compared to the general population,^43^ also on the basis of acromegaly medical treatment.^16^^,^^36^ The present study observed significant correlations between CBCT-derived indices and measures of bone quality. Specifically, CTI(S) and CTMI were significantly correlated with the TBS, indicating the potential in assessing bone quality in acromegalic patients, while the CTMI also showed a significant correlation with lumbar bone mineral density (BMD), suggesting its utility in evaluating bone density in this group.

Glucocorticoid (GC) excess, whether exogenous in patients on corticosteroids or endogenous, has notable effects on bone mass and fracture risk.^10^^,^^44^^,^^45^ The main mechanism of changes in bone metabolism in patients with CS is represented by the inhibition of bone formation. BMD at the level of the lumbar spine is significantly lower in these patients, but not at the hip or femoral neck, compared to patients without CS. In addition, TBS is also significantly lower in CS patients.^46^ In the present study, patients with CS were included as part of the secondary endocrine causes group. The analysis revealed that CTMI and CTI(S) showed significant correlations with TBS, while CTMI also demonstrated a significant correlation with lumbar BMD.

CTMI and CTI(S) indices show variability with bone quality, with higher indices generally indicating better bone quality. CTI(I) values are less variable across different bone qualities but show slightly higher averages in secondary endocrine causes. Postmenopausal women exhibit generally lower CBCT indices compared to patients with secondary endocrine causes, suggesting different bone density and quality profiles between these groups. The results suggest that CBCT-derived indices (CTMI, CTI(I), and CTI(S)) can effectively differentiate between varying levels of bone density and quality in both postmenopausal women and patients with secondary endocrine causes of low bone mass.

In postmenopausal women, all CBCT indices showed strong, significant correlations with bone health parameters, indicating robust utility in this group. In patients with secondary endocrine causes, correlations were generally weaker and less consistent, with significant correlations mainly observed with TBS scores and some BMD measures for CTMI and CTI(S). Overall, CBCT indices are more effective in assessing bone health in postmenopausal women compared to those with secondary endocrine causes, but they still offer some value in the latter group. As in the other 2 endocrine pathologies, in CS the CTI(S) and CTMI were significantly correlated with the TBS. Given the accelerated bone loss in CS,^10^ CTI(S) can help in early detection of deteriorating bone quality, allowing early therapeutic intervention and, as a result, a stable dental implant site.

CBCT indices (CTMI, CTI(I), and CTI(S)) have a stronger predictive value for assessing bone quality (TBS) in postmenopausal women compared to patients with secondary endocrine causes of low bone mass. Significant associations were found for all 3 indices in postmenopausal women, suggesting that CBCT can be a reliable tool for evaluating bone quality in this group. However, the lack of significant associations in the secondary endocrine group suggests that CBCT indices may not be as effective in predicting bone quality for these patients. Additionally, neither group showed significant associations between CBCT indices, and the diagnosis of osteoporosis as defined by lumbar or femoral neck *T*-scores or by AACE criteria, indicating that while CBCT is useful for assessing bone quality, it may not be sufficient on its own for diagnosing osteoporosis across different patient populations. These findings highlight the need for further research to explore the utility of CBCT in patient groups with different diseases and for comprehensive osteoporosis assessment.

The studied CBCT indices have inconsistent predictive value across the 3 endocrine conditions associated with low bone mass. While none of the indices demonstrated significant predictive value for osteoporosis in patients with diabetes mellitus or CS, there was a notable exception in acromegaly patients, where CTMI showed a significant association with osteoporosis prediction. This variability suggests that the effectiveness of CBCT indices in predicting osteoporosis may depend on the specific underlying endocrine disorder. Although a limitation of this study is the relatively small sample size of the groups when dividing the secondary causes and the relatively small sample with osteoporosis in this group, CS and acromegaly are rare pathologies.

Specialists in fields such as implantology and maxillofacial surgery use CBCT to assess the pre-implant bone support, as well as for post-intervention monitoring. In the field of endodontics, CBCT provides information about the corono-radicular morphology and the presence of accessory root canals.^47^ Using CBCT images, the exact size of periapical inflammatory processes can be calculated, which are difficult to estimate with conventional imaging techniques. CBCT is used to investigate the exact location of pathologies in the jaw, such as tumours, inflammatory lesions, and the precise location of impacted teeth.^48^^,^^49^  *In vitro* studies have demonstrated the usefulness of evaluating bone mass at the level of the mandibular medial condyle to predict the risk of osteoporosis, with a moderate correlation between this parameter and the mandibular osteoporosis index.^50^ Additionally, the same *in vitro* studies have shown that CBCT can quantify trabecular bone structures quite accurately, demonstrating a significant correlation with microCT assessments performed for the same purpose.^39^^,^^51^

The purpose of pre-implant CBCT evaluation is to identify patients at risk for unfavourable dental implant prognosis due to low bone mass or impaired bone microarchitecture and to treat them prior to the implant in order to increase the chances of post-implant success, for example with anabolic agents such as teriparatide.^24^ Regarding the radiation impact, although a single CBCT scan can replace all orthodontic radiographs, one set of radiographs still involves 2-4 times less radiation than a CBCT. Depending on the scanning mode, the radiation dose of a CBCT is approximately 3-6 times that of a digital panoramic radiograph, 8-14 times that of a postero-anterior cephalogram, and 15-26 times that of a lateral cephalogram. Additionally, to fully reconstruct cephalograms, including the cranial base and other critical structures, the CBCT portrait mode must be used, which further highlights the difference in radiation exposure (131.7 vs 35.81 μSv). Shielding radiation-sensitive organs can significantly reduce the effective dose.^52^

CBCT-derived measurements offer a non-invasive method to assess bone health, making it easier to monitor patients over time without the need for frequent DXA scans. This study underscores the value of panoramic CBCT-derived indices, particularly the CT mandibular index superior (CTI(S)) and the CT mental index (CTMI), in assessing bone density and quality across various patient populations with low bone mass, including postmenopausal women and individuals with endocrine disorders such as diabetes mellitus, acromegaly, and CS. The results suggest that CBCT-derived indices (CTMI, CTI(I), and CTI(S)) can effectively differentiate between varying levels of bone density and quality in both postmenopausal women and patients with secondary endocrine causes of low bone mass.

## Conclusion

Statistically significant correlations were highlighted between the CBCT indices and both quantitative (BMD, *T*-score) and qualitative (TBS) bone mass parameters in both the groups. Regarding BMD, the correlations had better predictive power in the postmenopausal group rather than in the endocrine diseases group, with a few exceptions, such as the case of the CTMI index in acromegaly. The CTMI, CTI(S), and CTI(I) indices have predictive value for bone quality assessed with TBS in both the groups. In the group of postmenopausal women, all 3 CBCT indices showed strong correlations with DXA parameters. In the group of patients with secondary endocrine causes of low bone mass, CTMI and CTI(S) significantly correlated with the TBS score, and CTMI also showed a significant correlation with lumbar BMD.

Performing CBCT before the implant procedure helps identifying patients with low bone mass or decreased bone quality and treat them, accordingly, thus improving the stability and the prognosis of the implant.
